# GABA, Glutamate, and NAA Levels in the Deep Cerebellar Nuclei of Essential Tremor Patients

**DOI:** 10.3389/fneur.2021.664735

**Published:** 2021-05-06

**Authors:** Arthur W. G. Buijink, Naomi Prent, Nicolaas A. Puts, Anouk Schrantee, Wouter V. Potters, Anne-Fleur van Rootselaar

**Affiliations:** ^1^Department of Neurology and Clinical Neurophysiology, Amsterdam University Medical Centers, Amsterdam Neuroscience, University of Amsterdam, Amsterdam, Netherlands; ^2^Department of Forensic and Neurodevelopmental Sciences, Sackler Institute for Translational Neurodevelopment, Institute of Psychiatry, Psychology, and Neuroscience, King's College London, London, United Kingdom; ^3^Department of Radiology and Nuclear Medicine, Amsterdam University Medical Centers, University of Amsterdam, Amsterdam, Netherlands

**Keywords:** tremor, gamma-aminobutyric acid, ^1^H-magnetic resonance spectroscopy, essential tremor, cerebellum

## Abstract

**Background:** Essential tremor is among the commonly observed movement disorders in clinical practice, however the exact pathophysiological mechanisms underlying tremor are unknown. It has been suggested that Purkinje cell alterations play a causal factor in tremorgenesis. Altered levels of inhibitory (GABA) and excitatory (glutamate+glutamine, Glx) neurotransmitters could be markers for Purkinje cell alterations. We hypothesize that GABA and Glx levels in the dentate nuclei could be differentially altered in patients responsive to either anticonvulsants or β-adrenergic blockers.

**Methods:** In this explorative study in patients with essential tremor, we measured gamma-aminobutyric acid (GABA) and glutamate+glutamine (Glx) levels in the dentate nucleus region using ^1^H-magnetic resonance spectroscopy (MRS) in seven patients using propranolol, five patients using anticonvulsants, and eight healthy controls.

**Results:** There were no group differences with respect to GABA+/Cr, Glx/Cr, NAA/Cr, and GABA+/Glx ratios. There was no correlation with tremor severity.

**Discussion:** Our results are in line with previously published studies; however, additional studies on a larger number of patients are warranted to confirm these findings. Furthermore medication-subgroups did not exhibit differences with respect to GABA+/Cr, Glx/Cr, NAA/Cr, and GABA+/Glx ratios. A recent study, of similar size, found an inverse association between tremor severity and the GABA+/Glx ratio in the cerebellum of essential tremor patients. We were unable to replicate these findings. The field of tremor research is plagued by heterogeneous results, and we would caution against drawing firm conclusions based on pilot studies.

## Introduction

A prevalent hypothesis on the pathophysiology of essential tremor (ET) suggests Purkinje cell pathology as a causal factor in tremorgenesis. Purkinje cells form the sole output from the cerebellar cortex, and lead to the deep cerebellar nuclei, including the dentate nucleus. Several previous studies showed Purkinje cell alterations, decreased cerebellar cortical N-acetyl-L-aspartate (NAA) levels supporting neurodegenerative processes, and decreased numbers of gamma-aminobutyric acid (GABA) receptors in the dentate nucleus in patients with ET (1–4). The hypothesis that altered levels of inhibitory (GABA) and excitatory (glutamate+glutamine, Glx) neurotransmitters could be a marker for Purkinje cell loss could not be confirmed in previous ^1^H-magnetic resonance spectroscopy (MRS) studies, which showed no differences between ET and control subjects (5, 6). These studies suggest that the lack of observed differences could be due to compensatory terminal sprouting of Purkinje cells (6). A recent study, however, did show an inverse association between tremor severity and cerebellar GABA+/Glx ratio (5). The relevance of this observation is debatable, since GABA+/Cr and Glx/Cr ratios did not show group differences in this study.

Several treatments for ET currently exist, of which propranolol, primidone, topiramate, and gabapentin have a level A or B recommendation, based on small studies (7). The mechanism of action of β-adrenergic blockers like propranolol is unknown. Anticonvulsants might act through ion channel and gamma-aminobutyric-acid (GABA) receptor modulation (8). Interestingly, response to primidone is not a predictor for response to propranolol (9). A consensus paper on ET research suggested characterizing ET subtypes based on medication response (10). We hypothesize that GABA and Glx levels in the dentate nuclei could be differentially altered in ET patients responsive to either anticonvulsants or β-adrenergic blockers. In this explorative study, our aim was to assess whether subtyping ET based on medication use might provide differences in GABA and Glx levels in the dentate nucleus region in specific subgroups. We will compare ET patients using either propranolol or anticonvulsants, and healthy controls.

## Materials and Methods

### Participants

Patients were either recruited through a website of our research group, referred by neurologists from other hospitals or through our research database. Patients were selected based on criteria for ET defined by the Consensus Statement on the Classification of Tremors (11), and the use of anticonvulsant medication (GABA group) or propranolol (PROP group). Patients with characteristics of ET plus were excluded. Other exclusion criteria were a score <26 on the Mini-Mental State Examination, neurological disorders (for patients: other than essential tremor), age < 18 years, the use of medication affecting the CNS and magnetic resonance-related contra-indications. Patients tapered their anti-tremor medication following a personalized scheme to allow for proper washout based on the half-life of the specific preparations. Measurements took place after four half-lives had elapsed, ensuring a subtherapeutic remaining fraction of 1/16. Patients were videoed following a strict protocol based on The Essential Tremor Rating Assessment Scale (TETRAS) (12). Reviewing these videos, blinded to treatment group, tremor severity was assessed OFF medication by an experienced rater (A.W.G.B.) using TETRAS parts A and B (12). For practical reasons, tremor severity could not be assessed while on medication. Healthy controls, also fulfilling the criteria above, were matched for age, gender and handedness. Data was anonymized. The study was carried out in accordance with the Declaration of World Medical Association (13) and was approved by the Medical Ethical Committee of the Academic Medical Center, Amsterdam.

### Data Acquisition

**Figure 1:**
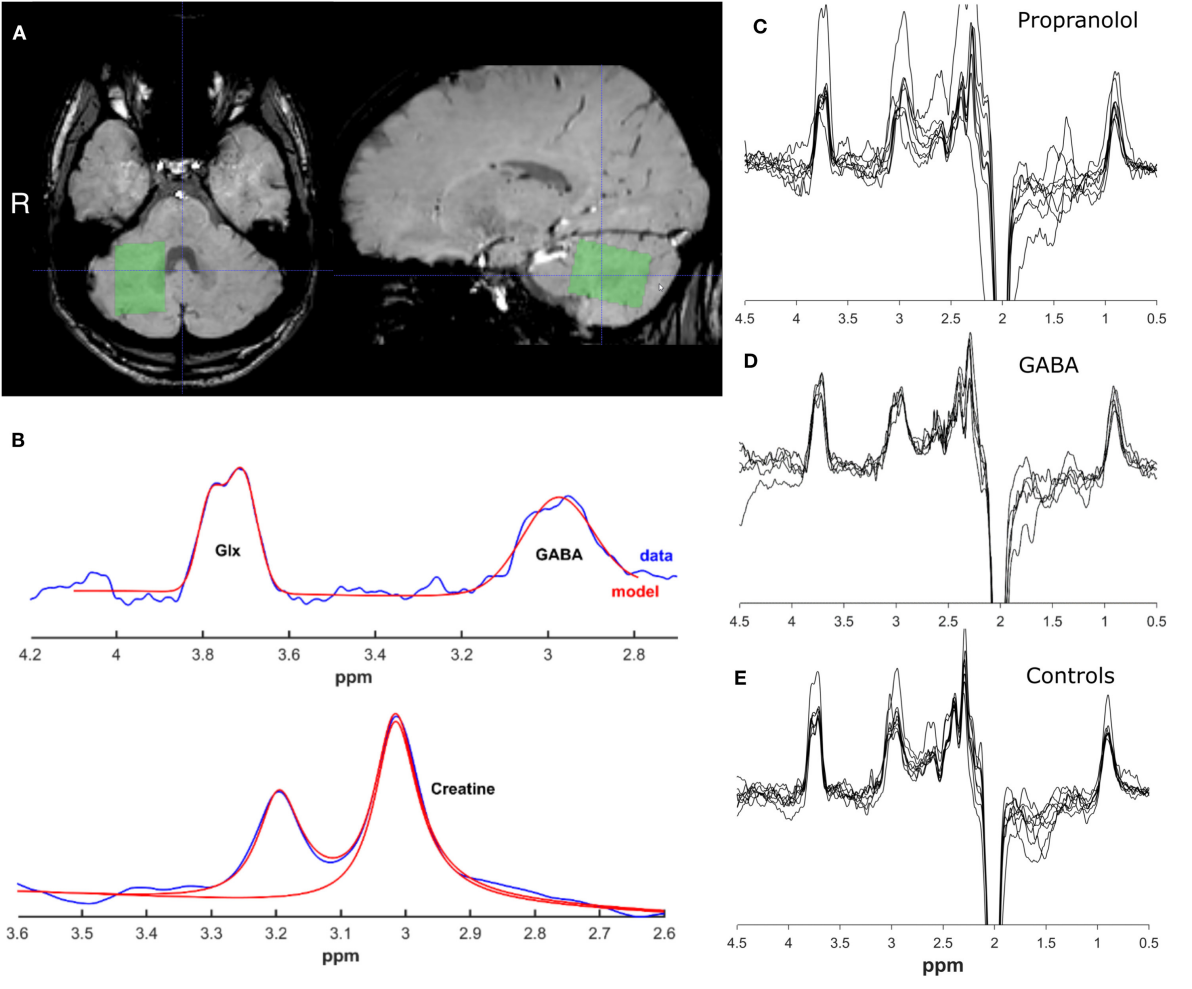
Representative voxel placement and spectra. **(A)** Location of MRS voxel on a T2*–weighted image. **(B)** Example spectra and fitted model for creatine, GABA+ and Glx peak range. Images were created using GANNET toolbox v3.0. **(C)** All spectra for propranolol group **(D)**. All spectra for GABA group **(E)**. All spectra for control group.

All participants underwent a magnetic resonance spectroscopy (MRS) scan session, in which GABA levels were assessed in a single voxel in the right deep cerebellar nuclei region. Data were acquired using a 3.0 T Philips MR Scanner (Philips Medical Systems, Best, The Netherlands), using a 32-channel receive-only head coil and body coil transmission. The anatomical T1-weighted image was obtained with the following scan parameters: TR/TE = 9.0/3.7ms, flip angle 8°, FOV = 256 mm × 256 mm × 170 mm, voxel size = 1.0 mm × 1.0 mm × 1.0 mm. The T2^*^–weighted image was obtained with the following scan parameters: TR/TE = 25.7/21.8 ms, flip angle 17°, FOV = 213 mm × 216 mm × 130 mm, voxel size = 1.0 mm × 1.0 mm × 1.1 mm. J-difference edited MRS spectra were acquired using a MEGA-PRESS sequence from a 2.5 cm × 2.5 cm × 2.5 cm voxel in the right deep cerebellar nuclei region (as seen on the T2^*^ image) with the following parameters: TR/TE = 2,000/68 ms, dynamic scans = 320 (2 × 160 ON and OFF), 14 ms editing pulses placed at 1.9 ppm (ON) and 7.46 ppm (OFF) with 1,024 data points and 2 kHz spectral width, for an ~10 min acquisition. The voxel was placed manually and centered on the characteristic hypointensity in the deep cerebellar nuclei region as seen on T2^*^ images, and angled to contain as little CSF as possible (Figure 1A).

### Image Analysis

Edited MRS spectra were analyzed using the Gannet GABA analysis toolbox ([version 3.0 (14)], Figure 1B). Phasing, apodization, and frequency correction were performed automatically in this toolbox. GABA+ and Glx levels were calculated according to standard procedures, as described in detail elsewhere. In short, the time-domain data is processed into a frequency-domain GABA+ and Glx-edited spectrum. Using a nonlinear, least-squares fitting, the GABA+ and Glx level at respectively 3 and 3.75 ppm are estimated. The assessment of GABA+ using MEGA-PRESS however results in co-editing of macro-molecules such as proteins (because the editing pulse at 1.9 ppm is known to co-edit macromolecule signals at 1.7 ppm), which contribute to the edited GABA peak at 3.0 ppm and are therefore referred to as GABA+ levels. GABA levels are quantified against the creatine signal. GABA+ fit errors were calculated with the Gannet GABA analysis toolbox to assess the data quality of the spectra. The SPM12 toolbox (version 3) was used in MATLAB (The Mathworks, Natick, MA) to co-register the T1-weighted scan to the MRS scan in the Gannet toolbox. Using the segment function of SPM12, the T1-weighted image was segmented to determine the tissue fractions (gray matter, white matter, and CSF) for the voxel. The Gannet toolbox estimates the CSF-corrected GABA+ values based on these tissue fractions (14). Exclusion criteria for bad data quality were based on visual inspection of the GABA+ edited difference spectrum, frequency drifts of the residual water spectrum, the creatine signal before and after frequency and phase correction, and the fit of the GABA+, the water and creatine signal, in addition to quantitative measurements of the provided fit error and expected full-width/half-maximum of the signal peaks, and on visual inspection of the voxel position.

### Statistical Analysis

MATLAB was used for all statistical analyses. Considering the small sample size, non-parametric tests were chosen. Wilcoxon rank-sum tests were performed to compare GABA+/Cr-levels, Glx/Cr-levels, GABA/Glx levels and NAA/Cr levels between medication subgroups of ET (GABA and PROP), and to compare each patient group to healthy controls. Spearman's rank correlation coefficients were calculated between MRS output measures and TETRAS-scores OFF-medication. *P* < 0.05 were considered statistically significant. Because the probability of type I error cannot be decreased without increasing the probability of type II error, such that real differences may not be detected, no correction for multiple comparisons was applied for this pilot study. There are no available formal sample size criteria for MRS studies. Considering the recent study by Tapper and colleagues (5), and the exploratory nature of this study, a similar sample size was chosen.

## Results

### Demographical and Clinical Characteristics

After screening, 26 participants were eligible for inclusion. In retrospect two patients did not meet the inclusion criteria of the Consensus Statement on the Classification of Tremor based on the video recordings. Four cases were excluded because of poor data quality. Five ET patients responsive to anticonvulsants, seven ET patients responsive to propranolol medication and 8 healthy controls were included in the final analysis. See Figures 1C–E for all MRS spectra. Demographical and clinical characteristics are presented in Table 1. For characteristics per patient including specific medication use and previously tried tremor-medication see Supplementary Table 1.

**Table 1:** Demographical data of total included subjects (*n* = 20) and subject groups.

	**Total**	**GABA**	**PROP**	**HC**
*n*	20	5	7	8
Males (%)	14 (70%)	4 (80%)	4 (67%)	6 (75%)
Age in years (SD)	62.2 (12.2)	69.8 (3.3)	55.9 (11.6)	63.0 (14.1)
Age at onset (SD)	37.5 (21.2)	36.6 (29.1)	38.14 (16.00)	N/A
Disease duration (SD)	24.2 (19.6)	33.2 (26.3)	17.71 (11.32)	N/A
Familial tremor (%)	10 (83%)	4 (80%)	6 (86%)	N/A
Alcohol sensitivity (%)	7 (60%)	2 (40%)	5 (71%)	N/A
TETRAS (SD)	17.7 (8.9)	24.70 (8.68)	12.64 (4.93)	N/A
Head tremor (%)	5 (42%)	3 (60%)	2 (29%)	N/A
Tremor medication	N/A	Primidone (*n* = 3)	Propranolol (*n* = 7)	N/A
		Gabapentin (*n* = 2)		

*Data are mean (SD) or n (%). Subject groups: GABAergic medication (GABA), propranolol medication (PROP), healthy controls (HC). N/A, not applicable. TETRAS, The Essential Tremor Rating Assessment Scale. For full characteristics per patient including specific medication use and previously tried tremor-medication see Supplementary Table 1*.

### MRS Results

**Figure 2:**
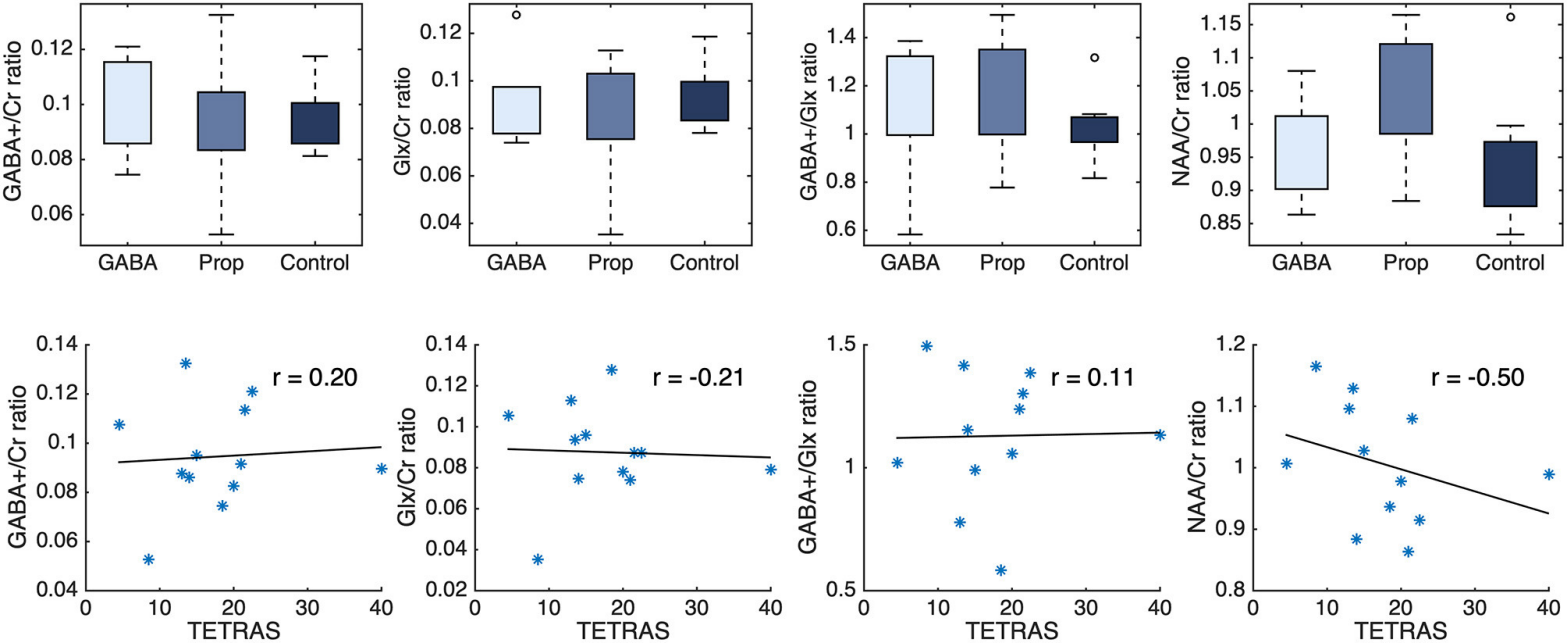
**(Upper)** GABA+/Cr, Glx/Cr, GABA+/Glx, and NAA/Cr ratios show no difference between ET patients using GABAergic-medication (GABA) or propranolol medication (Prop), and healthy controls (Control). **(Lower)** TETRAS-scores (OFF-medication) of ET patients are not correlated with GABA+s/Cr, Glx/Cr, GABA+/Glx, and NAA/Cr ratios. *represent individual ET patients. Cr, creatine; ET, Essential Tremor; Glx, glutamate plus glutamine.

There was no significant difference between healthy controls (*n* = 8), patients responsive to propranolol (*n* = 7, PROP) and patients responsive to anticonvulsants (*n* = 5, GABA) regarding GABA+/Cr, Glx/Cr, GABA+/Glx, and NAA/Cr ratios (Figure 2). For GABA+/Cr ratio: PROP vs. controls [W] = 54, *p* = 0.87, GABA vs. controls [W] = 38, *p* = 0.72, PROP vs. GABA [W] = 36, *p* = 0.64. For Glx/Cr ratio: PROP vs. controls [W] = 53, *p* = 0.78, GABA vs. controls [W] = 34, *p* = 0.94, PROP vs. GABA [W] = 32, *p* = 1.00. For GABA+/Glx ratio: PROP vs. controls [W] = 72, *p* = 0.07, GABA vs. controls [W] = 44, *p* = 0.22, PROP vs. GABA [W] = 34, *p* = 0.88. For NAA/Cr ratio: PROP vs. controls [W] = 63, *p* = 0.46, GABA vs. controls [W] = 39, *p* = 0.62, PROP vs. GABA [W] = 23, *p* = 0.15. For details of all measurements, frequency drift, signal-to-noise-ratios, and full width at half maximum please see Supplementary Table 2. TETRAS-scores (OFF-medication) were not correlated with GABA+/Cr (*r* = 0.20, *p* = 0.53), Glx/Cr (*r* = −0.20, *p* = 0.51), GABA+/Glx (*r* = 0.10, *p* = 0.75), and NAA/Cr ratios (*r* = −0.50, *p* = 0.10) in ET (combined groups, *n* = 12).

## Discussion

This explorative study suggests that GABA+, Glx, and NAA levels within the dentate nucleus region are not different in ET compared to healthy controls. Additionally, medication-subgroups did not exhibit differences in metabolites of interest. As mentioned previously, two earlier studies provided similar results, where no differences in GABA+ and Glx levels were detected (5, 6). An additional study compared GABA+ levels between ET patients using primidone or no primidone during MRS measurements, and found no effect of concurrent primidone use on GABA concentrations (15). The more recent study by Tapper et al. did observe a small but statistically significant inverse association between GABA+/Glx ratios and tremor severity. In this study, 10 ET cases and 6 healthy controls were included. Voxel size was similar to our study (25 mm × 25 mm × 25 mm vs. 35 mm × 25 mm × 25 mm), however, we have only included MRS spectra of the right deep cerebellar nuclei region due to limited available scanning time, in contrast with inclusion of both right and left side in the study by Tapper et al. Tremor rating scales were different between studies, and are not directly comparable. GABA+ as measured using MRS is an indirect marker of neurotransmitter levels, reflecting cellular pools of GABA, but is also composed of macromolecules and homocarnosine (16). Glx is the combined signal of glutamate and glutamine, which cannot be separated using this technique. In addition to its role as a neurotransmitter, glutamate plays an extremely important role in energy metabolism, and glutamine is predominantly metabolic (16). Thus, GABA+ and Glx do not merely reflect “inhibition” or “excitation.” Moreover, these signals are noisy, and the GABA+/Glx ratio is therefore even more noisy, which makes the interpretation of this ratio complicated. It is debatable whether conclusions about pathophysiological mechanisms can be based on this ratio, especially when the GABA+/Cr and Glx/Cr ratios do not show group differences. Further research in this area is needed.

A major limitation of this study is its small sample size. Small group sizes and selection based on current medication use might have caused a type II error. However, the overlapping distributions per subgroup indicate that differences, if any, would be small. As already mentioned, the study by Tapper et al. did observe a statistically significant difference in GABA+/Glx ratios between ET patients and controls. It is worth mentioning that low power also increases the risk of type I error, reducing the likelihood that a statistically significant result reflects a true effect (17). The fact that results regarding GABA+ and Glx levels are in accordance with both previous MRS studies supports our results. Another limitation is the difference in mean age between the PROP and GABA group. Exact matching of subjects based on age was not feasible in the current setting. Although essential tremor is a common disorder, many patients have some (minor) additional symptoms (ET plus). We have used very strict inclusion criteria with respect to the disorder, co-morbidity, medication use and ability to undergo an MR-scan. A previous study did not find an age effect when looking at GABA levels corrected for voxel composition (18). We have used the same method of correction for voxel composition in our study.

In this explorative study, we confirm a previously identified lack of differences in GABA+ and Glx levels in the dentate nucleus region of essential tremor patients quantified with MRS (5, 6). Medication-subgroups did not exhibit differences in this respect. Furthermore, we could not replicate a previously observed association between GABA+/Glx ratios and tremor severity. MRS is a valuable technique in assessing metabolite changes within the tremor network. However, replication studies are needed before further conclusions can be drawn on the pathophysiological basis of these changes. The field of tremor research is plagued by heterogeneous results, and we would caution against drawing firm conclusions based on pilot studies.

## Data Availability Statement

The raw data supporting the conclusions of this article will be made available by the authors, without undue reservation.

## Ethics Statement

The studies involving human participants were reviewed and approved by Medical Ethical Committee of the Academic Medical Center, Amsterdam. The patients/participants provided their written informed consent to participate in this study.

## Author Contributions

AB and A-FR set up the study. AB performed data collection, interpreted the data, and wrote the initial manuscript. NP and WP performed data collection and analyzed the data. NAP and AS provided technical assistance and interpreted the data. All authors contributed in writing the manuscript. All authors read and approved the final manuscript.

## Conflict of Interest

The authors declare that the research was conducted in the absence of any commercial or financial relationships that could be construed as a potential conflict of interest.
