# Supplementary material for: GABA, Glutamate, and NAA Levels in the Deep Cerebellar Nuclei of Essential Tremor Patients
Source: Front Neurol. 2021 May 6;12:664735. doi: 10.3389/fneur.2021.664735 (PMC8136412; doi:10.3389/fneur.2021.664735)
Supplement: Supplementary file 1 [file Table_1.DOCX]

Supplementary Material

# Supplementary table 1. *Demographical and clinical characteristics of the included ET patients*

| **Pt** | **Age (yrs)** | **Sex** | **Disease duration (yrs)** | **FH** | **Alcohol resp.** | **TETRAS** | **Head tremor** | **Current meds** | **Previous meds** |
| --- | --- | --- | --- | --- | --- | --- | --- | --- | --- |
| 1 | 49 | M | 9 | + | + | 13.5 | + | Prop. 40 mg daily | No effect prim. and metoprolol |
| 2 | 54 | M | 38 | + | unk | 8.5 | - | None | Positive effect prop. |
| 3 | 40 | F | 12 | + | + | 14 | - | Prop. 10 mg prn | None |
| 4 | 56 | F | 26 | + | + | 15 | + | Prop. 10 mg prn | None |
| 5 | 68 | M | 5 | + | + | 13 | - | Prop. 80 mg CR qd | None |
| 6 | 74 | F | 20 | - | - | 20 | - | Prop. 10 mg t.i.d. | None |
| 7 | 50 | M | 14 | + | - | 4.5 | - | Prop. 10 mg prn | None |
| 8 | 71 | M | 51 | - | + | 21.5 | + | Prim. 75 mg prn | No effect prop. |
| 9 | 65 | M | 60 | + | unk | 22.5 | + | None | Positive effect gabapentin. No effect prop. |
| 10 | 72 | M | 7 | + | + | 40 | + | None | Positive effect prim. No effect prop. |
| 11 | 68 | F | 45 | + | - | 21 | - | Gabapentin 300 mg t.i.d. | No effect prop. |
| 12 | 73 | M | 3 | + | - | 18.5 | - | Prim. 25 mg t.i.d. | Prop. not tried due to contra-indication |

Abbreviations: *CR* = controlled release, *F* = female, *FH* = family history, *M* = male, *meds* = medication, *prn* = *pro re nata* (as needed), *prop* = propranolol, *prim* = primidone, *pt* = patient, *qd* = *quaque die* (once a day), *TETRAS* = total score on the The Essential Tremor Rating Assessment Scale*. t.i.d.:* Three times daily. *Unk* = unkown, *yrs* = years.

**Supplementary table 2.** *MRS results of total included subjects (n = 20) and subject groups.*

|  | **Total** | **GABA** | **PROP** | **HC** |
| --- | --- | --- | --- | --- |
| *n* | 20 | 5 | 7 | 8 |
| GABA+/Cr^a^ | 9.06∙10${}^{-2}$ (1.94∙10${}^{-2})$ | 9.15∙10${}^{-2}$ (2.96∙10${}^{-2})$ | 8.77∙10${}^{-2}$ (2.10∙10${}^{-2})$ | 9.09∙10${}^{-2}$ (1.47∙10${}^{-2})$ |
| Glx/Cr^a^ | 8.67∙10${}^{-2}$ (2.21∙10${}^{-2})$ | 8.72∙10${}^{-2}$ (1.96∙10${}^{-2})$ | 9.36∙10${}^{-2}$ (2.76∙10${}^{-2})$ | 8.61∙10${}^{-2}$ (1.63∙10${}^{-2})$ |
| GABA+/Glx^a^ | 1.06∙10^0^ (2.79∙10${}^{-1})$ | 1.24∙10^0^ (3.27∙10${}^{-1})$ | 1.06∙10^0^ (3.52∙10${}^{-1})$ | 1.04∙10 ^0^  (1.03∙10${}^{-1})$ |
| NAA/Cr^a^ | 9.63∙10${}^{-1}$ (1.58∙10${}^{-1})$ | 9.37∙10${}^{-1}$ (1.10∙10${}^{-1})$ | 1.03∙10^0^ (1.35∙10${}^{-1})$ | 9.11∙10${}^{-1}$ (9.71∙10${}^{-2}$) |
| GMF | 5.91∙10${}^{-1}$ (6.92∙10${}^{-2}$) | 5.61∙10${}^{-1}$ (8.47∙10${}^{-2}$) | 5.84∙10${}^{-1}$ (9.38∙10${}^{-2}$) | 6.01∙10${}^{-1}$ (2.87∙10${}^{-2}$) |
| WMF | 3.83∙10${}^{-1}$ (6.11∙10${}^{-2}$) | 3.75∙10${}^{-1}$ (6.46∙10${}^{-2}$) | 3.87∙10${}^{-1}$ (1.08∙10${}^{-1}$) | 3.83∙10${}^{-1}$ (2.00∙10${}^{-2}$) |
| CSFF | 2.94∙10${}^{-2}$ (2.24∙10${}^{-2}$) | 3.60∙10${}^{-2}$ (3.51∙10${}^{-2}$) | 2.91∙10${}^{-2}$ (1.08∙10${}^{-2}$) | 1.94∙10${}^{-2}$ (2.66∙10${}^{-2}$) |
| Frequency drift^b^ | 2.74∙10${}^{-2}$ (4.33∙10${}^{-2}$) | 6.49∙10${}^{-3}$ (5.87∙10${}^{-2}$) | 9.63∙10${}^{-3}$ (5.96∙10${}^{-2}$) | 3.12∙10${}^{-2}$ (1.87∙10${}^{-2}$) |
| FWHM  GABA+  Glx  NAA  Cr | 1.88∙10${}^{1}$ (3.50∙10${}^{0}$)  1.64∙10${}^{1}$ (1.29∙10${}^{0}$)  1.04∙10${}^{1}$ (1.23∙10${}^{0}$)  9.79∙10${}^{0}$ (1.39∙10${}^{0}$) | 2.09∙10${}^{1}$ (2.06∙10${}^{0}$)  1.62∙10${}^{1}$ (2.47∙10${}^{0}$)  1.09∙10${}^{1}$ (1.57∙10${}^{0}$)  9.84∙10${}^{0}$ (9.19∙10${}^{-1}$) | 1.86∙10${}^{1}$ (3.55∙10${}^{0}$)  1.69∙10${}^{1}$ (4.25∙10${}^{0}$)  1.06∙10${}^{1}$ (1.74∙10${}^{0}$)  9.94∙10${}^{0}$ (1.55∙10${}^{0}$) | 1.77∙10${}^{1}$ (2.50∙10${}^{0}$)  1.64∙10${}^{1}$ (1.03∙10${}^{0}$)  1.02∙10${}^{1}$ (1.03∙10${}^{0}$)  8.93∙10${}^{0}$ (1.57∙10${}^{0}$) |
| SNR  GABA+  Glx  NAA  Cr | 1.67∙10${}^{1}$ (4.51∙10${}^{0}$)  1.94∙10${}^{1}$ (4.99∙10${}^{0}$)  1.93∙10${}^{2}$ (4.52∙10${}^{1}$)  2.02∙10${}^{2}$ (5.18∙10${}^{1}$) | 1.45∙10${}^{1}$ (5.40∙10${}^{0}$)  1.87∙10${}^{1}$ (6.28∙10${}^{0}$)  1.92∙10${}^{2}$ (5.31∙10${}^{1}$)  2.04∙10${}^{2}$ (4.16∙10${}^{1}$) | 1.69∙10${}^{1}$ (9.21∙10${}^{0}$)  2.11∙10${}^{1}$ (7.64∙10${}^{0}$)  1.91∙10${}^{2}$ (4.48∙10${}^{1}$)  1.90∙10${}^{2}$ (4.21∙10${}^{1}$) | 1.72∙10${}^{1}$ (2.92∙10${}^{0}$)  1.94∙10${}^{1}$ (5.19∙10${}^{0}$)  2.12∙10${}^{2}$ (5.44∙10${}^{1}$)  2.36∙10${}^{2}$ (8.72∙10${}^{1}$) |
| Fit error  GABA+  Glx  NAA  Cr | 6.58∙10${}^{0}$ (3.50∙10${}^{0}$)  5.74∙10${}^{0}$ (3.17∙10${}^{0}$)  2.45∙10${}^{0}$ (6.72∙10${}^{-1}$)  1.46∙10${}^{0}$ (2.58∙10${}^{-1}$) | 5.72∙10${}^{0}$ (3.30∙10${}^{0}$)  5.19∙10${}^{0}$ (2.42∙10${}^{0}$)  2.44∙10${}^{0}$ (3.21∙10${}^{-1}$)  1.33∙10${}^{0}$ (1.22∙10${}^{-1}$) | 7.94∙10${}^{0}$ (4.50∙10${}^{0}$)  7.08∙10${}^{0}$ (5.34∙10${}^{0}$)  2.48∙10${}^{0}$ (8.27∙10${}^{-1}$)  1.49∙10${}^{0}$ (3.28∙10${}^{-1}$) | 6.58∙10${}^{0}$ (3.33∙10${}^{0}$)  5.81∙10${}^{0}$ (3,16∙10${}^{0}$)  2.40∙10${}^{0}$ (8.07∙10${}^{-1}$)  1.55∙10${}^{0}$ (2.31∙10${}^{-1}$) |

Subject groups: GABA medication (GABA), propranolol medication (PROP), healthy controls (HC). Data are median (IQR). ^a^ = CSF-corrected. ^b^: average frequency offset from Cr peak (ppm). Abbreviations: *Cr* = creatine, *CSF* = cerebrospinal fluid, *CSFF* = cerebrospinal fluid voxel fraction, *FWHM* = Full width at half maximum, *Glx* = glutamate plus glutamine, *GMF* = grey matter voxel fraction, *IQR* = Interquartile range, *NAA* = N-acetyl-L-aspartate, *SNR* = Signal to Noise Ratio, *WMF* = white matter voxel fraction.
